# Acute outcomes of cryoballoon vs. circular vs. pentaspline pulsed-field ablation catheters in combined pulmonary vein isolation and roof line ablation

**DOI:** 10.1007/s10840-025-02078-9

**Published:** 2025-05-29

**Authors:** Joerg Yogarajah, Julie Hutter, Patrick Kahle, Philipp Beaujean, Marko Tomic, Andreas Hain, Samuel Sossalla, Thomas Neumann, Malte Kuniss

**Affiliations:** 1 Department of Cardiology, Kerckhoff Heart Center, Campus Kerckhoff, Justus Liebig University Giessen, 61231 Bad Nauheim, Germany; 2 Department of Cardiology, Medical Clinic I, Justus Liebig University Giessen, 35392 Giessen, Germany

**Keywords:** Atrial fibrillation, Pulsed-field ablation, Cryoballoon, Pulmonary vein isolation, Left atrial roof ablation

## Abstract

**Background:**

Single-shot ablation systems are widely used for pulmonary vein isolation (PVI) in atrial fibrillation (AF). The use of novel pulsed-field ablation (PFA) systems enables ablation beyond PVI, such as left atrial roof ablation (LARA), which may improve outcomes in persistent AF.

**Objective:**

This study aimed to compare the acute efficacy, feasibility, and safety of PVI combined with LARA using three different single-shot ablation systems in patients with persistent AF and left atrial enlargement undergoing their first AF ablation.

**Methods:**

Consecutive patients undergoing PVI with LARA using cryoballoon or PFA systems were included. Baseline characteristics, procedural parameters, and complication rates were assessed.

**Results:**

We included 125 patients with persistent AF and left atrial dilation, divided into cryoballoon (*n* = 65), pentaspline PFA (*n* = 30), and circular PFA (*n* = 30) groups. Acute PVI was achieved in 100% of veins. Fewer applications were required for LARA with cryoballoon vs. PFA (4 vs. 8 vs. 10, *P* < 0.001). Conduction block was confirmed in 95%, 100%, and 100% of patients (*P* = 0.421). Procedural times were longer with cryoballoon (87.0 vs. 64.0 vs. 68.0 min, *P* < 0.001), but fluoroscopy times were shorter (12.2 vs. 15.3 vs. 15.1 min, *P* = 0.002). Contrast medium use was higher in the cryoballoon group (*P* < 0.001). Adverse events were rare and predominantly minor, with three complications in the cryoballoon group and one in the PFA groups (*P* = 0.493).

**Conclusion:**

All single-shot ablation systems demonstrated comparable efficacy and safety for PVI and LARA, with differences in procedural feasibility. Further and larger studies are needed.

**Graphical Abstract:**

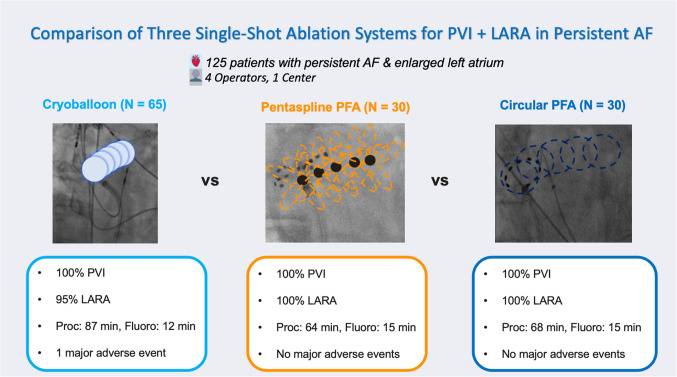

## Introduction

Single-shot ablation systems are increasingly being adopted as a first-line approach for pulmonary vein isolation (PVI). Various systems, including cryoballoon (CB) and pulsed-field ablation (PFA), are available. Notably, these advanced technologies enable ablation beyond PVI, which may further contribute to reducing atrial arrhythmia recurrence.

CB ablation is a well-established single-shot ablation system for the treatment of paroxysmal and persistent atrial fibrillation (AF) [1, 2]. Emerging evidence suggests that CB ablation beyond PVI, particularly through adjunctive left atrial roof ablation (LARA) in dilated left atria, may provide clinical benefits for patients with persistent AF [3–7]. These findings highlight the potential value of expanding ablation strategies in this challenging patient population.

Meanwhile, PFA represents a groundbreaking, non-thermal energy source for the ablation of AF. Its hallmark feature is the selective targeting of myocardial tissue, minimizing collateral damage to adjacent structures compared to traditional methods such as CB or radiofrequency (RF) ablation [8]. While PFA offers a favorable safety profile and reduced procedural durations, its long-term efficacy appears comparable to established modalities [9]. Currently, multiple PFA systems are under development, with two major single-shot PFA systems already in clinical use: the FARAPULSE™ system (Boston Scientific, Marlborough, MA), a pentaspline PFA catheter, and the PulseSelect™ system (Medtronic, Minneapolis, MN), a circular array PFA catheter [10, 11].

Both systems have consistently demonstrated efficacy in achieving PVI, high safety, and feasibility outcomes. However, data on performing non-PV ablations—such as posterior wall isolation (PWI), roof-line, and cavo-tricuspid isthmus ablation—using both PFA systems, especially with the circular array PFA catheter, are rare [11–14].

Moreover, despite significant advancements in ablation technology, comparative data between PFA systems and CB ablation remain limited, particularly in the context of non-PV ablations for persistent AF. This study aimed to evaluate the acute efficacy, feasibility, and safety of PVI combined with LARA using three distinct single-shot ablation systems (CB vs. circular array vs. pentaspline PFA catheter) in patients undergoing their first ablation for persistent AF.

## Methods

### Study population

This single-center observational study included consecutive patients with persistent AF and left atrial (LA) enlargement (LA area > 20 cm^2^) who underwent their first catheter ablation with PVI and LARA. The procedures were performed using either a CB catheter, a circular array PFA system, or a pentaspline PFA system between November 2023 and October 2024. During the data collection period, CB and PFA were performed in an alternating fashion, aiming for a balanced distribution between both modalities. Within the PFA group, the FARAPULSE™ system was used between November 2023 and April 2024, followed by the PulseSelect™ system from April to September 2024, depending on availability. All procedures were performed by operators experienced in single-shot catheter ablation for AF. Patients were recruited from routine clinical practice at the Kerckhoff Heart Center, presenting with an indication for AF ablation. Exclusion criteria included the presence of intracavitary thrombus, prior left atrial ablation, severe valvular heart disease, or pregnancy.

The study was approved by the Ethics Committee of Justus Liebig University of Giessen (approval no. AZ 83/24) and adhered to the principles of the Declaration of Helsinki. All participants were thoroughly informed about the procedure, including potential ablation beyond PVI, and provided written informed consent prior to treatment.

### Preprocedural management

Intracardiac thrombus was excluded in all patients via transesophageal echocardiography prior to ablation. Transthoracic echocardiography was performed pre-procedurally to evaluate the LA area and left ventricular ejection fraction (LVEF). LA enlargement was assessed using the LA area measured in cm^2^, as this is the standard parameter used in our clinical routine. An LA area > 20 cm^2^ was defined as enlarged [5].

For patients on novel oral anticoagulants, the medication was paused for at least 12 h before the procedure. In contrast, patients receiving vitamin K antagonists continued anticoagulation therapy with an international normalized ratio target of 2–3.

### Ablation procedure

All ablation procedures were carried out under either deep sedation or general anesthesia, with four primary operators performing the interventions. All procedures were performed under fluoroscopic guidance without the use of a 3D electroanatomical mapping system. No additional radiofrequency ablation was performed in any of the cases.

A 10-pole diagnostic catheter was inserted into the coronary sinus. After a single transseptal puncture was achieved using the Brockenbrough technique (BRK-1) and a transseptal sheath (SL-1), heparin was administered to maintain an activated clotting time exceeding 300 s. An exchange wire was advanced into the left superior pulmonary vein (LSPV), enabling the replacement of the transseptal sheath with either a 12 Fr steerable sheath (FlexCath Advance™, Medtronic, Minneapolis, MN, USA), a 13 Fr steerable sheath (FARADRIVE, Boston Scientific, Marlborough, MA) or a 10 Fr steerable sheath (FlexCath Contour™, Medtronic, Minneapolis, MN, USA). PV angiography was then performed to delineate the anatomies of the left atrium and PVs.

In CB ablation procedures, a 28-m CB ballon (Arctic Front Advance Pro™, Medtronic) with the inner lumen spiral mapping catheter (Achieve Advance™ catheter, 20 mm diameter, Medtronic) was positioned via a steerable sheath in the LA and advanced into the PV to record electric PV signals. The CB was inflated proximal to the PV ostium and pushed against it. PV occlusion was documented by injection of contrast medium into the PV. During ablation, PV signals were recorded. Cryothermal energy was applied for 180 s per freeze cycle, following a time-to-isolation guided ablation protocol. If time-to-isolation was not achieved within 60 s, the freeze cycle was extended to the full 240 s. One or more bonus freezes were applied if the PV was not isolated after the first freeze cycle. During CB ablation along septal PVs, phrenic nerve capture was monitored by diaphragmatic contraction, tactile feedback, and diaphragmatic compound motor action potentials using a diagnostic catheter positioned in the superior vena cava.

The pentaspline PFA catheter procedure involved advancing the 12 F multielectrode PFA catheter (FARAWAVE, Boston Scientific) into the left atrium via a steerable sheath. To facilitate accurate positioning at the PV ostium, a guidewire (InQuire, Merit Medical Systems, Inc., South Jordan, UT) was placed in the target PV. At each PV, at least eight pulses were delivered, with four performed in the “basket” configuration and four in the “flower” configuration. For optimal circumferential ablation, the catheter was rotated 36° following the first two pulses in each configuration. Energy delivery was executed in a biphasic waveform, independent of the cardiac rhythm. Each application consisted of five consecutive pulse trains.

For cases utilizing the circular PFA catheter, the over-the-wire PFA catheter (PulseSelect™, Medtronic) was introduced into the left atrium via a steerable sheath and positioned within the PVs to record electrical PV signals. Catheter positioning was verified through fluoroscopic imaging. The ablation catheter is designed with a 9 Fr shaft featuring bidirectional steering and a 25-mm loop equipped with nine electrodes capable of sensing, pacing, and ablating. A minimum of four antral and four ostial applications were delivered per vein. Each application comprised four biphasic, bipolar pulse trains. To ensure complete PVI and address potential gaps between electrodes 1 and 9, the catheter was circumferentially rotated into four different positions after each application.

To safeguard against phrenic nerve injury, a low-voltage electrical pulse was delivered from all nine electrodes to test for phrenic nerve capture, both before and after PFA applications in the right-sided PVs with a circular PFA system. In cases using the pentaspline PFA catheter, phrenic nerve capture was monitored with a diagnostic catheter after ablation.

**Fig. 1:**
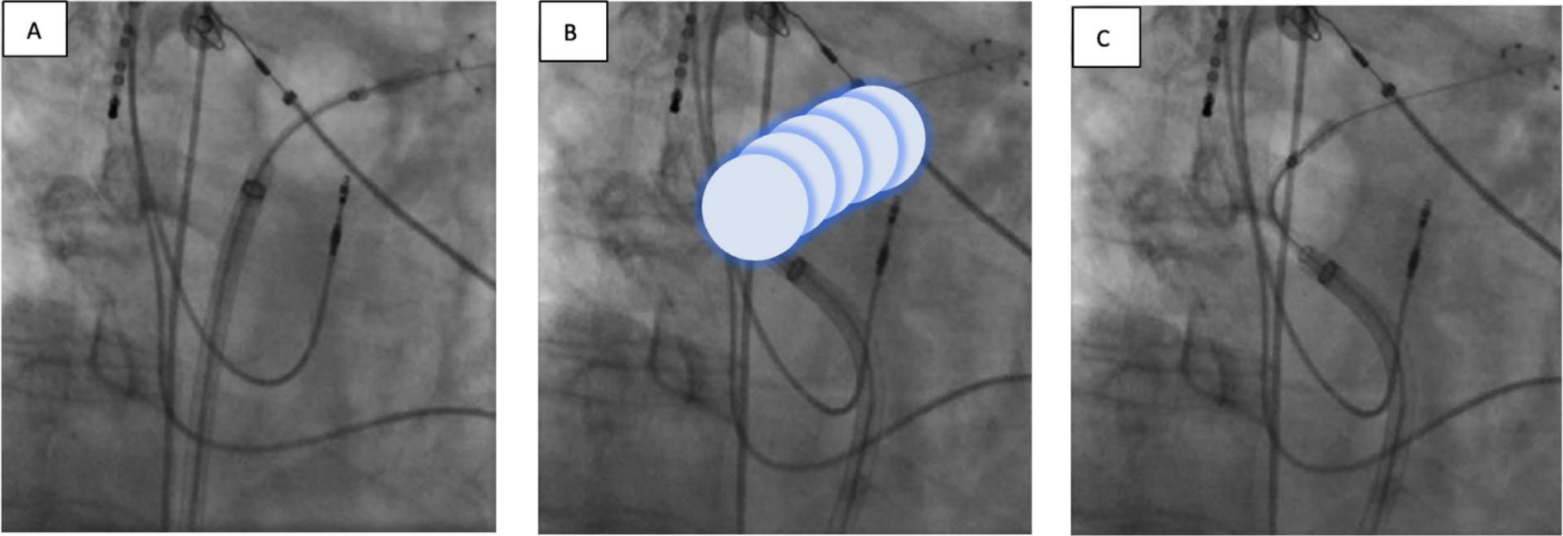
Maneuvers for cryoballoon left atrial roof ablation in right anterior oblique (RAO) view. **A** First freeze. **B** Incremental cryoballoon advancement by slight clockwise rotation and slight sheath retraction. **C** Last freeze

In the CB group, additional LARA was performed after PVI. The Achieve catheter was deeply positioned in the LSPV (Fig. 1). Starting near the LSPV isolation position, sequential overlapping 150 s freezes were performed along the LA roof. The CB was incrementally repositioned by slight clockwise rotation with slight sheath retraction until the isolation position for the right superior pulmonary vein (RSPV) was reached [4].

**Fig. 2:**
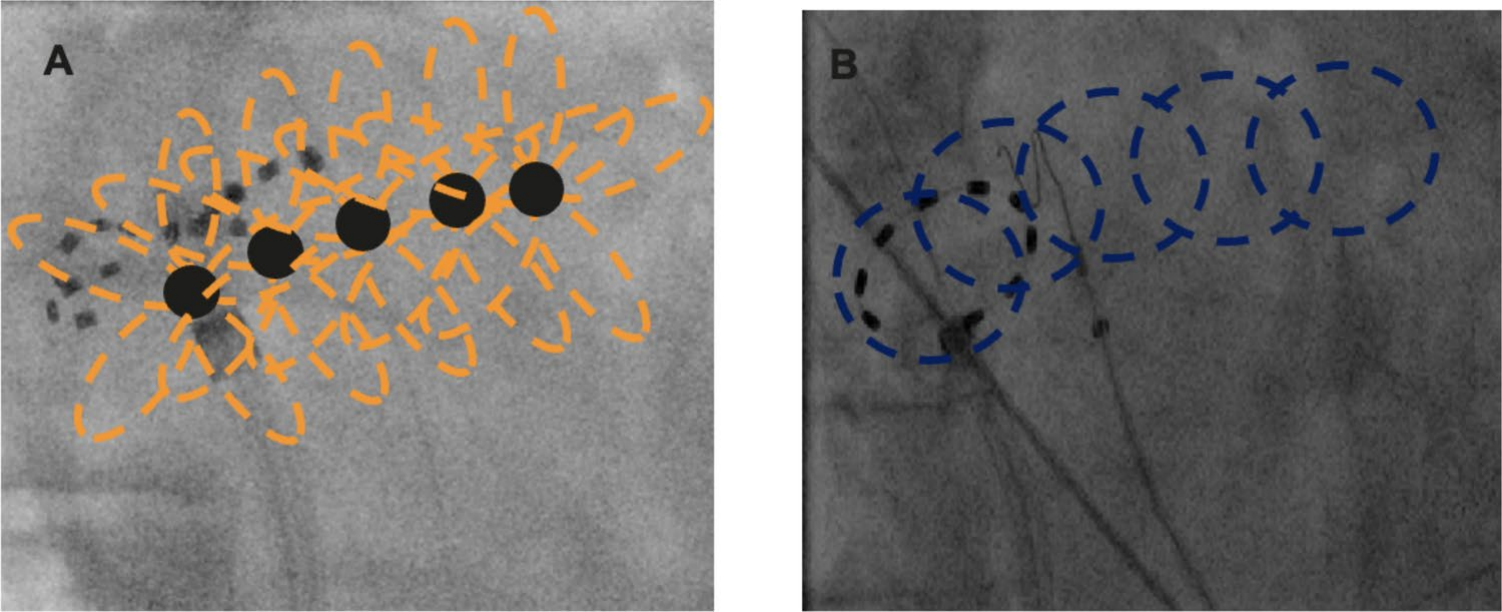
Left atrial roof ablation using pulsed-field ablation in right anterior oblique (RAO) view. **A** Applications performed with a pentaspline pulsed-field ablation catheter. **B** Applications performed with a circular pulsed-field ablation catheter

In PFA patients undergoing additional LARA following PVI, the PulseSelect™ or FARAWAVE™ catheter (in “flower” position) was positioned in the LSPV (Fig. 2). Sequential overlapping energy applications were delivered along the LA roof, beginning near the LSPV isolation site. The catheter was gradually repositioned by retracting the sheath and incrementally rotating the catheter until it reached the isolation site for RSPV [13].

The procedural endpoint was defined as complete PVI and conduction block across the LA roof, verified in sinus rhythm. For patients who exhibited persistent AF after the initial ablation, electrical cardioversion was performed. Upon successful restoration to sinus rhythm, pulmonary vein entrance and exit blocks as well as LA roof conduction block were confirmed. All assessments were performed as part of the standard workflow, without predefined observation intervals.

Bidirectional conduction block across the LA roof was assessed using differential pacing.

The Achieve or PFA catheter was positioned at both the caudal and cranial regions of the posterior LA wall. Baseline pacing in sinus rhythm was conducted at the right atrial upper septum with a cycle length of 500 ms. Activation times were measured at the caudal and cranial posterior LA wall regions near the LA roof. LA roof conduction block was confirmed by observing caudocranial ascending activation patterns at the posterior LA wall and a conduction delay exceeding 150 ms adjacent to the ablation site [4, 13].

### Postprocedural management

Immediately after ablation, a transthoracic echocardiography was performed to rule out pericardial effusion. Patients were monitored by telemetry until discharge the next day.

Oral anticoagulation was reinitiated at least 3 h after the procedure. It was continued for at least 2 months and thereafter according to CHA2DS2-VA Score. Proton pump inhibitors were prescribed in all patients for 6 weeks. Antiarrhythmic drug administration was discontinued immediately after ablation.

### Endpoints and follow-up

The primary endpoint was acute procedural success, defined as the complete isolation of all PVs, verified through both entry and exit block, alongside bidirectional linear anatomical block of any additional ablation lesions. Secondary endpoints included procedural metrics, such as total procedure duration, fluoroscopy time, and dose-area product.

The primary safety endpoint focused on identifying predefined complications associated with the system and procedure, occurring either during the intervention or throughout the hospital stay.

### Statistical analysis

Continuous variables are expressed as mean ± standard deviation or as median with interquartile range, depending on data distribution. Categorical variables are reported as absolute numbers and percentages. Group comparisons were performed using Student’s *t*-test or analysis of variance for continuous variables with a normal distribution, while the Mann–Whitney *U* test or Kruskal–Wallis test was applied for nonparametric data. For categorical variables, comparisons were made using Pearson’s chi-square test or Fisher’s exact test, as appropriate. Statistical calculations were performed with the statistical analysis software R (R Version 4.2.0, 2022).

## Results

### Baseline characteristics

A total of 125 consecutive patients undergoing PVI and LARA were included in this study. The cohort was divided into three groups based on the ablation system used: CB group (*n* = 65), pentaspline PFA group (*n* = 30), and circular PFA group (*n* = 30). Baseline characteristics are summarized in Table 1. All patients presented with persistent AF and LA enlargement. The majority were male and exhibited preserved LVEF. Patients in the PFA groups, particularly those in the circular PFA group, were older (*P* = 0.018) and had a higher prevalence of coronary artery disease (*P* = 0.029). Additionally, the pentaspline PFA group demonstrated a slightly lower body mass index (BMI) compared to the other groups (*P* = 0.042). No statistically significant differences were observed among the groups for other baseline parameters.

**Table 1:** Baseline characteristics

Patient characteristics	Cryoballoon catheter *N* = 65	Pentaspline PFA catheter *N* = 30	CircularPFA catheter *N* = 30	*P* value
Female gender, *n*	14 (21%)	11 (37%)	6 (20%)	0.178
Age, years	65 (60–72)	67 (62–70)	74 (64–75)	0.018
Persistent AF, *n*	65 (100%)	30 (100%)	30 (100%)	1.000
BMI, kg/m^2^	30.0 (26.6–33.0)	27.1 (25.0–30.7)	30.5 (26.8–33.6)	0.042
CHA2DS2 VA	2 (1–3)	2 (1–3)	3 (2–3)	0.109
Duration since first AF diagnosis, months	21 (7–65)	10 (5–24)	8 (5–21)	0.065
Left atrial area, cm^2^	26.9 (22.9–30.0)	24.0 (21.8–26.0)	25 (23.02–29.3)	0.055
Left ventricular ejection fraction, %	54.1 (55.0–60.0)	55.0 (52.5–60.0)	60.0 (50.0–60.0)	0.833
Congestive heart failure, *n*	15 (23%)	5 (17%)	7 (24%)	0.800
Hypertension, *n*	47 (72%)	22 (73%)	23 (76%)	0.941
Diabetes mellitus, *n*	12 (18%)	4 (13%)	6 (20%)	0.805
Obstructive sleep apnea, *n*	6 (9%)	1 (3%)	3 (10%)	0.507
Coronary artery disease, *n*	7 (11%)	6 (20%)	11 (36%)	0.029
Chronic obstructive pulmonary disease, *n*	3 (5%)	1 (3%)	1 (3%)	0.942
Asthma, *n*	3 (5%)	1 (3%)	0 (0%)	0.809
Pacemaker, *n*	0 (0%)	1 (3%)	0 (0%)	0.466
Implantable cardioverter defibrillator, *n*	1 (2%)	1 (3%)	0 (0%)	0.717
History of stroke/transient ischemic attack, *n*	7 (11%)	4 (14%)	4 (13%)	0.929

Data are expressed as median (interquartile range), or *n* (percentage)

*PFA *pulsed-field ablation, *AF *atrial fibrillation, *BMI *body mass index

### Procedural data

Procedural data are shown in Table 2. PVI was achieved in all patients. The number of freezes/PFA applications for PVs per patient was different between the groups: 4.5 (4.0–5.0) vs. 32.0 (32.0–34.0) vs. 35.0 (33.0–37.0), *P* < 0.001. Fewer applications of roof line ablation were performed in the CB group than in the PFA groups: 4 (4–5) vs. 8 (6–12) vs. 10 (8–12), *P* < 0.001. Bidirectional conduction block across the LA roof was confirmed in 62/65 patients in the CB group, 30/30 in pentaspline PFA, and 30/30 in the circular PFA group (*P* = 0.421).

**Table 2:** Procedural data

Procedural characteristics	Cryoballoon catheter *N* = 65	Pentaspline PFA catheter *N* = 30	Circular PFA catheter *N* = 30	*P* value
General anesthesia (intubation), *n*	0 (0%)	1 (3.3%)	2 (6.7%)	0.100
Deep sedation (no intubation), *n*	65 (100%)	29 (97%)	28 (93%)	0.100
Total number of isolated PVs, *n*	262/262 (100%)	119/119 (100%)	120/120 (100%)	1.000
Number of applications/freezes per patient (PVs), *n*	4.5 (4.0–5.0)	32.0 (32.0–34.0)	35.0 (33.0–37.0)	< 0.001
Number of applications/freezes per patient (LARA), *n*	4 (4–5)	8 (6–12)	10 (8–12)	< 0.001
Successful LARA, *n*	62 (95%)	30 (100%)	30 (100%)	0.421
Procedural time (skin-to-skin), min	87.0 (76.2–99.0)	64.0 (55.7–78.0)	68.0 (61.0–73.0)	< 0.001
Total LA-dwell time, min	72.5 (61.0–85.0)	44.0 (42.0–52.0)	47.0 (42.0–52.0)	< 0.001
LA-dwell time for LARA, min	12.5 (11.0–16.0)	6.0 (5.0–8.0)	6.5 (5.0–9.0)	< 0.001
Fluoroscopy time, min	12.2 (9.6–15.3)	15.3 (12.8–16.6)	15.1 (12.8–18.2)	0.002
Total area dose, Gycm^2^	5.5 (4.5–7.6)	6.1 (3.0–8.9)	8.4 (5.3–10.0)	0.176
Contrast medium, ml	31.5 (26.2–40.0)	20.0 (15.0–24.0)	18.0 (16.0–20.0)	< 0.001

Data are expressed as median (interquartile range) or *n* (percentage)

*PFA *pulsed-field ablation, *PVs *pulmonary veins, *LARA *left atrial roof ablation, *LA *left atrial

**Fig. 3:**
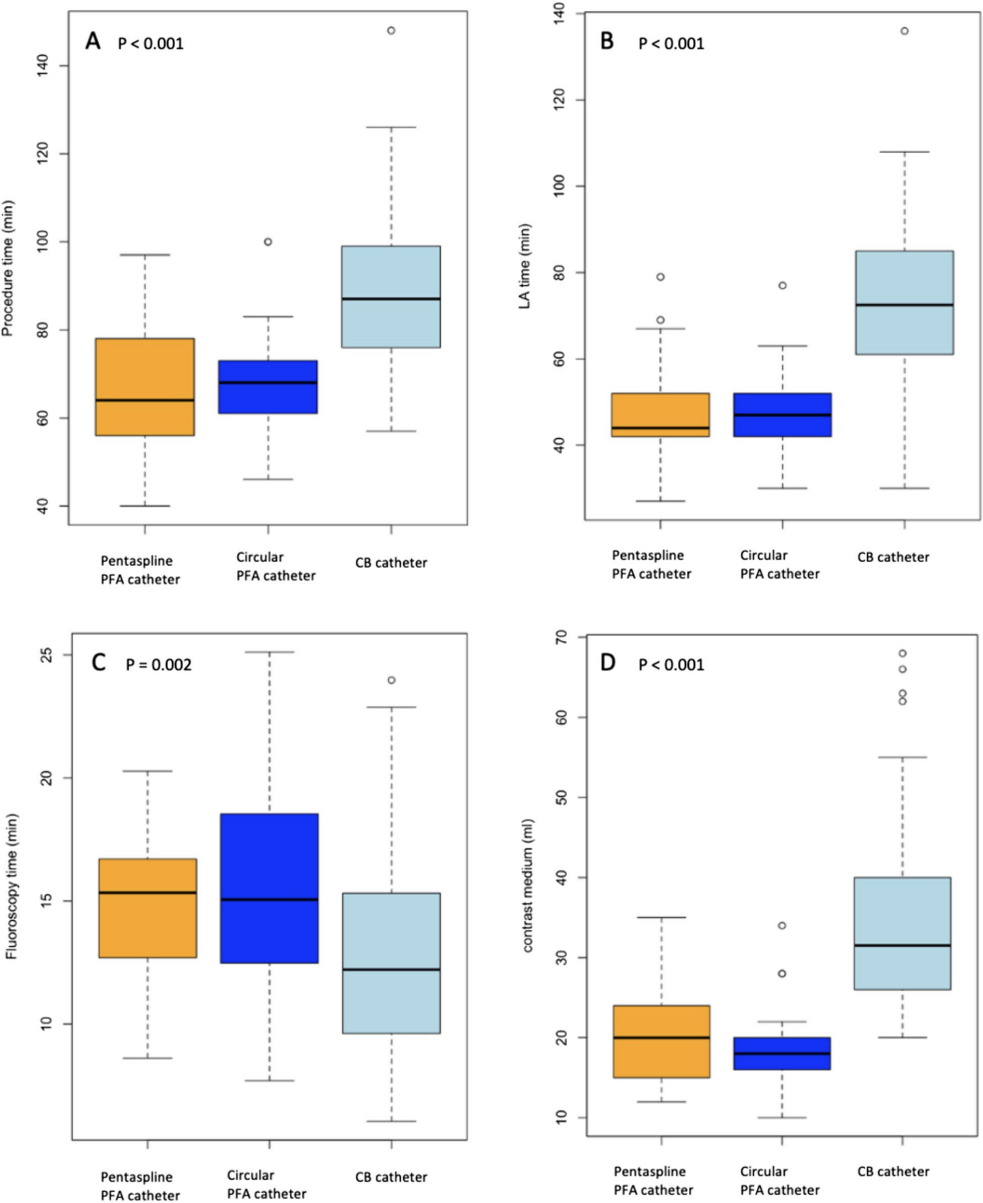
Procedural parameters. Comparison of procedural parameters between the 3 single-shot ablation systems for combined PVI and LARA. **A** Procedure time. **B** LA dwell time. **C** Fluoroscopy time. **D** Contrast medium use. PFA, pulsed-field ablation; LA, left atrial; CB, cryoballoon; PVI, pulmonary vein isolation; LARA, left atrial roof ablation

Procedural times were longer in CB compared to PFA groups, specifically with regard to total skin-to-skin time (87.0 (76.2–99.0) vs. 64.0 (55.7–78.0) vs. 68.0 (61.0–73.0) min, *P* < 0.001) and LA dwell time (72.5 (61.0–85.0) vs. 44.0 (42.0–52.0) vs. 47.0 (42.0–52.0) min, *P* < 0.001) (Fig. 3). Out of the total LA dwell time, the duration specifically required for roof-line ablation was 12.5 min (IQR 11.0–16.0) for cryoballoon, 6.0 min (IQR 5.0–8.0) for the pentaspline PFA catheter, and 6.5 min (IQR 5.0–9.0) for the circular array PFA system, *P* < 0.001. Fluoroscopy time was shorter in the CB group than in the PFA groups (12.2 (9.6–15.3) vs. 15.3 (12.8–16.6) vs. 15.1 (12.8–18.2) min, *P* = 0.002). The use of contrast medium was highest in the CB group (31.5 (26.2–40.0) vs. 20.0 (15.0–24.0) vs. 18.0 (16.0–20.0) ml, *P* < 0.001).

During the 1-day hospital stay, AF recurrence was observed in one patient from the pentaspline PFA group, requiring electrical cardioversion. Similarly, in the CB group, one recurrence of AF and one recurrence of atypical atrial flutter were observed, both managed with electrical cardioversion.

### Complications

The complication rate did not differ significantly between the three groups (*P* = 0.493). Major and minor complications are summarized in Table 3. In the CB group, 3 out of 65 patients experienced complications, including one sustained phrenic nerve palsy as the sole major complication, along with two minor vascular access issues. The circular PFA group reported one minor vascular complication, while no complications occurred in the pentaspline PFA group.

**Table 3:** Adverse events

Adverse events	Cryoballoon catheter *N* = 65	Pentaspline PFA catheter *N* = 30	Circular PFA catheter *N* = 30	*P* value
Major adverse events				
Pericardial tamponade, *n*	0	0	0	-
Coronary artery spasm, *n*	0	0	0	-
Cerebrovascular accident, *n*	0	0	0	-
Sustained phrenic nerve palsy, *n*	1 (1.6%)	0	0	0.628
Atroesophageal fistula, *n*	0	0	0	-
Thermal esophaegeal injury, *n*	0	0	0	-
Pulmonary vein stenosis, *n*	0	0	0	-
Major bleeding requiring transfusion, *n*	0	0	0	-
Vascular access complications requiring intervention, *n*	0	0	0	-
Death, *n*	0	0	0	-
Minor adverse events				
Pericardial effusion (no requiring intervention), *n*	0	0	0	-
Transient ischemic attack, *n*	0	0	0	-
Transient phrenic nerve palsy, *n*	0	0	0	-
Vascular access complications (no requiring intervention), *n*	2 (3.2%)	0	1 (3.4%)	0.614
Transient ST elevation, *n*	0	0	0	-

Data are expressed as *n* (percentage)

*PFA *pulsed-field ablation

Minor bleeding and hematoma in the CB group were managed with compression and a Femostop device, while a false aneurysm with an arteriovenous fistula was treated with compression bandages and thrombin injection. In the circular PFA group, an arteriovenous fistula resolved with pressure bandages.

No other major adverse events, such as esophageal injury, pericardial effusion, cerebrovascular accident, or death, occurred during the procedure or hospital stay. Conduction disturbances, ST-segment elevations, and coronary spasms were also absent.

## Discussion

### Main findings

This is the first report to directly compare the acute efficacy, feasibility, and safety of three single-shot ablation systems—CB, pentaspline PFA, and circular PFA—in patients with persistent AF and LA enlargement undergoing combined PVI and LARA.

All technologies demonstrated high efficacy, achieving a high rate of PVI and successful LARA. CB required fewer applications for LARA compared to both PFA systems, whereas PFA systems demonstrated shorter procedure times and reduced contrast medium usage. Fluoroscopy time was longer with the PFA systems. The complication rates were low and comparable across all three systems, suggesting that both CB and PFA systems are safe for combined PVI and LARA.

### Acute efficacy and feasibility

The rationale for incorporating LARA in patients with persistent AF and significant LA enlargement lies in the complexity of the underlying AF substrate. Persistent AF, especially when accompanied by LA dilation, is often linked to a widespread and diffuse fibrotic substrate that sustains arrhythmias despite successful PVI [15]. Addressing additional sites beyond the PVs, such as the LA roof, aims to modify the atrial substrate, potentially enhancing outcomes in this challenging patient population [5].

Several studies, primarily utilizing RF catheters, have reported varying levels of efficacy for additional ablation strategies [16–18]. However, data on CB and PFA ablation beyond PVI remain limited. Existing reports highlight the widespread adoption of integrated CB ablation approaches across various institutions. A few trials suggest that incorporating additional substrate modifications, such as LARA or PWI, using CB ablation may reduce atrial arrhythmia recurrence [3–5, 19–21]. Data on additional LARA using PFA for first AF ablation are limited. However, non-PV ablation targets, such as the posterior wall or superior vena cava, have been described [12, 13, 22].

In our study, all PVs were isolated with those three single-shot ablation systems. The acute success rate of LARA in our CB group (95%) aligns closely with previous findings [4, 5]. In our study, CB ablation required significantly fewer applications to achieve LARA compared to PFA systems. This is likely due to CB technology’s ability to create broader and potentially deeper atrial lesions with each application, in contrast to the ablation zones produced by a single PFA application. When comparing different trials, it is evident that fewer applications are necessary for LA substrate ablation using CB ablation compared to PFA, predominantly due to technical requirements [23–25].

The procedure and LA dwell time in the CB group were the longest, primarily due to the longer freeze duration (over 180 s for pulmonary veins and 150 s for a single freeze on the roof) (*P* < 0.001). The pentaspline PFA catheter exhibited slightly shorter procedural times compared to the circular PFA catheter, likely due to fewer applications required for both LARA and PVI (PVs 32 vs. 35, LARA 8 vs. 10 PFA applications). Nonetheless, fluoroscopy times were similar between the two PFA groups (15.3 vs. 15.1 min). This could potentially be attributed to the slightly higher BMI observed in the circular PFA group compared to the pentaspline PFA group [26].

The higher fluoroscopy times (*P* = 0.002) observed with PFA systems compared to CB system can be explained by the need for multiple applications and frequent catheter rotations for PVI and particularly for LARA, which prolongs imaging requirements. Conversely, CB ablation requires fewer applications and rotations, contributing to shorter fluoroscopy durations. Another contributing factor could be the extensive experience with the CB system in our center, which may have led to more efficient catheter handling and shorter fluoroscopy times compared to the PFA systems. The higher contrast medium usage in the CB group (31.5 vs. 20.0 vs. 18.0 ml) is due to the need for contrast injection to confirm pulmonary vein occlusion during the procedure. In contrast, during PFA procedures, contrast was used only for pulmonary vein angiography prior to ablation, resulting in lower overall usage.

Interestingly, in contrast to our fluoroscopy-guided ablation approach, other strategies have been described to optimize the procedure by reducing fluoroscopy time or minimizing the use of contrast medium. Recent reports and case series have demonstrated the feasibility of performing CB and PFA procedures without the use of fluoroscopy by relying on 3D electroanatomical mapping systems and intracardiac echocardiography [27, 28]. These findings suggest that completely fluoroscopy-free workflows may be achievable with all currently available single-shot ablation technologies. Moreover, some studies have described the assessment of pulmonary vein occlusion using real-time pressure waveform analysis as an alternative to contrast injection. This technique may help reduce contrast medium usage in CB procedures [29].

### Safety

In this study, complication rates were similar across the CB, pentaspline PFA, and circular PFA groups. Thermal-related complications, such as a phrenic nerve palsy in the CB group, were rare. Vascular complications occurred rarely, with two cases in the CB group and one in the pentaspline PFA group, none of which were attributed to the energy source. Notably, no cardiac tamponade, esophageal injury, or death occurred, underscoring the strong safety profiles of both cryoballoon and PFA-based ablation strategies.

Ablation beyond the PVs has been demonstrated to be safe in several studies. For instance, our group previously reported that LARA performed with CB technology in patients with persistent AF improved outcomes compared to PVI alone, without compromising safety [4, 5]. Moreover, Erkapic et al. demonstrated comparable esophageal safety outcomes between patients undergoing PVI alone and those receiving additional LARA [30].

Ablation beyond PVs with PFA, such as PWI, has shown exceptional safety across various studies [12, 22, 23]. Although coronary spasms have occasionally been reported with PFA systems, particularly when performing a mitral isthmus line or CTI line, our study found no such complications [8]. These findings further emphasize the favorable safety profile of PFA, especially for ablation strategies extending beyond the PVs, such as LARA.

Generally, the acute efficacy of PVI and LARA, as well as the safety profiles of all three ablation systems, was favorable, likely reflecting the extensive experience at this high-volume center with single-shot devices, particularly in performing PVI and ablation beyond PVs using CB.

### Clinical implications

This study provides critical real-world insights into the performance of three single-shot ablation systems—CB and two PFA catheters (pentaspline and circular)—in the context of combined PVI and LARA for persistent AF and left atrial enlargement. Notably, it highlights comparable acute efficacy and safety across these systems, while underscoring differences in procedural metrics.

These findings contribute to the growing body of evidence supporting the acute efficacy of single-shot devices, particularly PFA systems, in treating persistent AF with this ablation strategy. To build on these acute outcomes, future clinical research should prioritize long-term evaluations of PFA systems and that ablation strategy. This includes assessing freedom from AF recurrence, lesion durability for both PV and LARA, and comparing with thermal ablation methods.

### Study limitations

The findings are based on a single-center observational study with a relatively small patient cohort. Additionally, follow-up beyond hospital discharge was not available, limiting insights into mid- and long-term outcomes such as lesion durability and arrhythmia recurrence of this ablation strategy. However, long-term follow-up is still ongoing and will be reported separately after completion.

## Conclusions

This study demonstrates comparable high efficacy and safety profiles for the three single-shot ablation devices used in combined PVI and roof line ablation. CB required fewer applications for LARA, whereas PFA catheters were associated with shorter procedural and LA dwell times as well as reduced contrast medium usage, albeit with longer fluoroscopy times. Larger, long-term studies are warranted to further evaluate the outcomes of these ablation systems and strategies.

## Data Availability

The data presented in this study are available on request from the corresponding authors.
